# Supplementary material for: Unveiling Moroccan Nature’s Arsenal: A Computational Molecular Docking, Density Functional Theory, and Molecular Dynamics Study of Natural Compounds against Drug-Resistant Fungal Infections
Source: Pharmaceuticals (Basel). 2024 Jul 4;17(7):886. doi: 10.3390/ph17070886 (PMC11279552; doi:10.3390/ph17070886)
Supplement: Supplementary file 1 [file pharmaceuticals-17-00886-s001.zip › pharmaceuticals-3066476-supplementary.pdf]

Unveiling Moroccan Nature's Arsenal: A Computational Molecular Docking, Density Functional Theory, and Molecular Dynamics Study of Natural Compounds against Drug-Resistant Fungal Infections

Imane Yamari [1], Oussama Abchir [1], Hassan Nour [1], Meriem Khedraoui [1], Bouchra Rossafi [1], Abdelkbir Errougui [1], Mohammed Talbi [1], Abdelouahid Samadi [2,*], MHammed El Kouali [1], Samir Chtita [1,*]

[1] Faculty of Sciences Ben M'Sik, Hassan II University of Casablanca, Sidi Othman, Box 7955, Casablanca, Morocco

Department of Chemistry, College of Science, United Arab Emirates University, Al Ain P.O. Box 15551, United Arab Emirates

* Corresponding author: A.S. samadi@uaeu.ac.ae and S.C. samirchtita@gmail.com

Table S1: Comprehensive compilation of 2D structures for 297 natural compounds from diverse plant families.

Lamiaceae					
1 *origanum compactum*					
53	Borneol 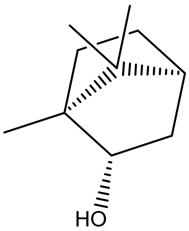	218	Ocimene 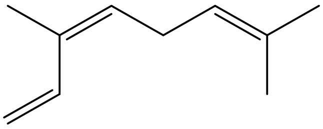	278	Thymol 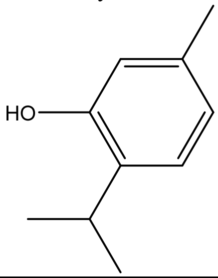
67	Carvacrol 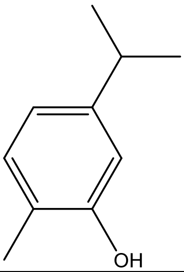	219	Octan-3-ol 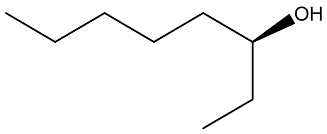	305	Alpha-phellandrene 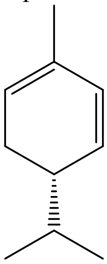

70	Caryophyllene oxide 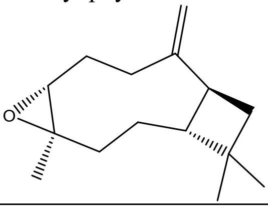	220	Octan-3-one 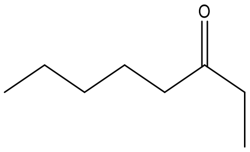	306	Alpha-pinene 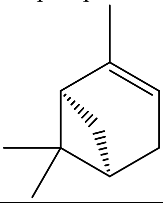
96	P-cymene 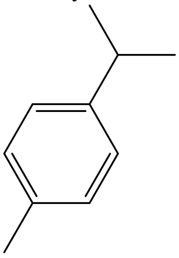	255	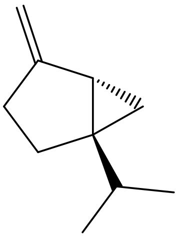 Sabinene	309	Alpha-pinene 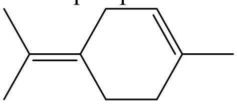
185	Caryophyllene 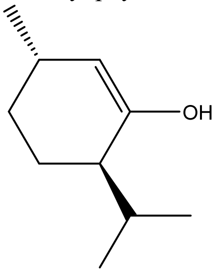	261	Santolina triene 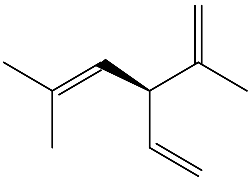	278	Alpha-terpinolene 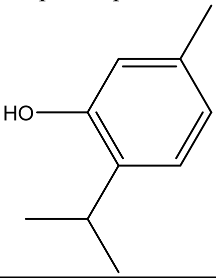
2 mentha pulegium					
9	2-cyclohexen-1 ol 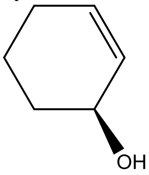	237	Piperitone 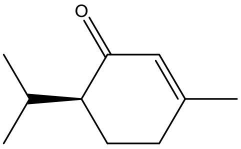	195	3-chloro-4-t-butyl-6-methylpyridazine 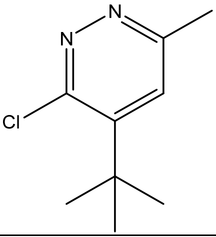
21	3-acetyl-2.5-dimethyl thiophene 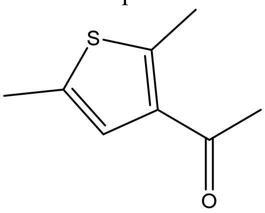	244	Pulegone 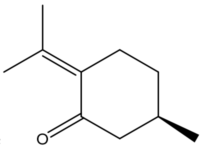	205	Neoisomenthol 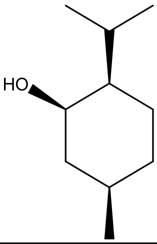

22	3-cyclopentyl propionic acid 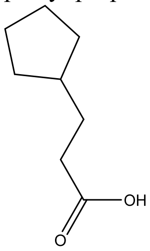	254	Rotundifolone 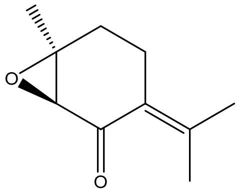	236	Piperitenone 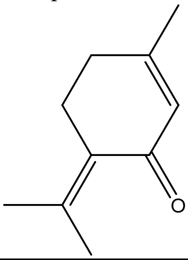
70	Caryophyllene oxide 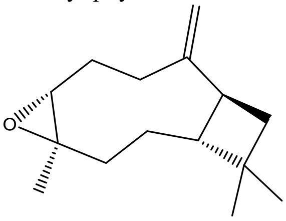	286	Undecatriene 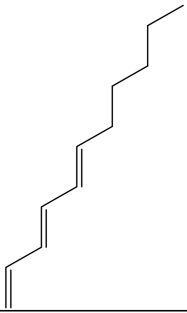	195	3-chloro-4-t-butyl-6-methyl pyridazine 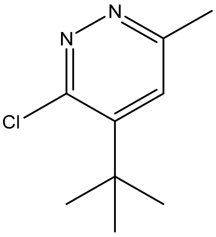
92	Cyclohexanol 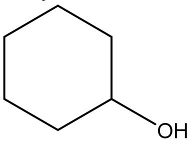	306	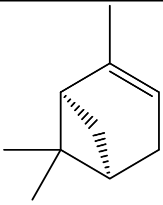 Alpha-pinene	188	Menthone 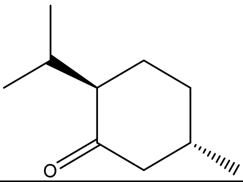
142	Glycocyanidine 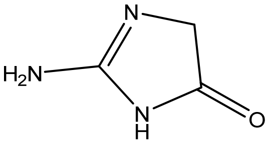	153	Humulene 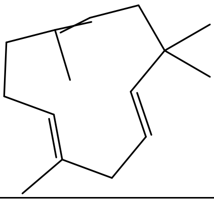	186	Menthene 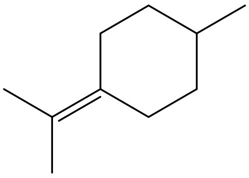
150	Hexahydrofarnesyl acetone 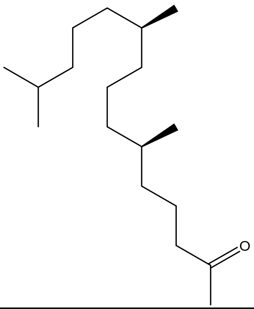	162	Isopulegone 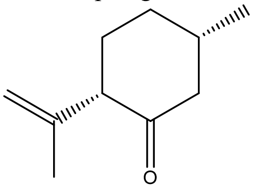	187	Menthol 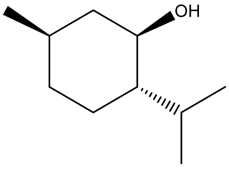
4 lavandula angustifolia					

53	Borneol<br>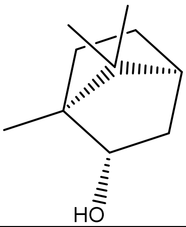	255	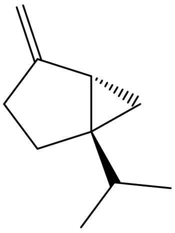<br>Sabinene	132	Geranyl acetate<br>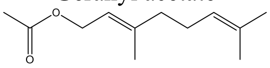
64	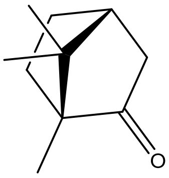<br>Camphor	274	Terpineol<br>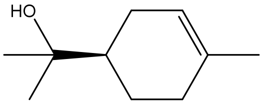	172	Linalyl acetate<br>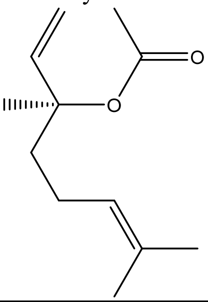
70	Caryophyllene, oxide<br>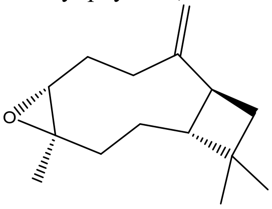	296	Alpha-bisabolol<br>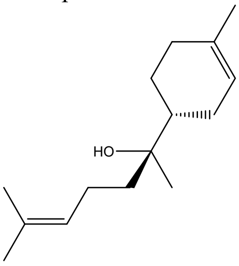	119	Farnesyl acetate<br>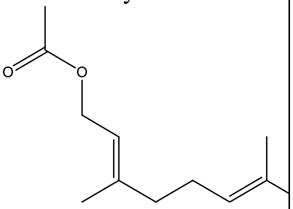
5 majorana hortensis moench					
63	Camphene<br>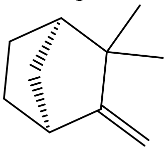	226	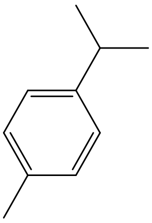<br>P-cymol	306	Alpha-pinene<br>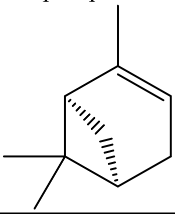
206	Neophytadiene<br>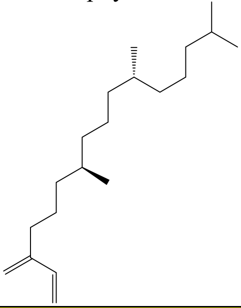	326	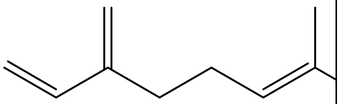<br>Beta-myrcene	309	Alpha-terpinolene<br>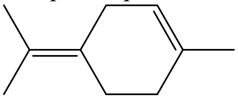
6 rosmarinus officinalis					

53	Borneol 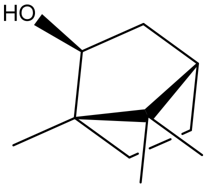	170	Linalol 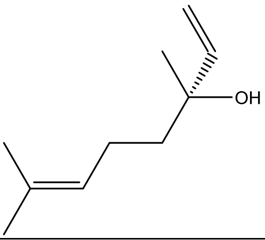	69	Caryophyllene 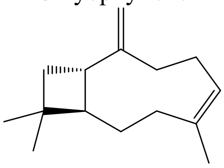
63	Camphene 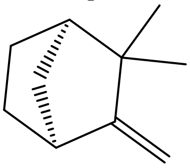	275	Terpinolene 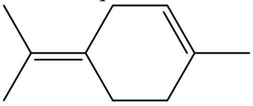	96	P-cymene 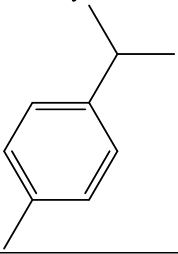
64	Camphor 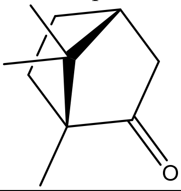	326	Beta-myrcene 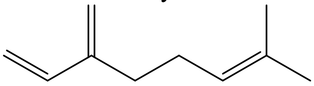	114	Eucalyptol 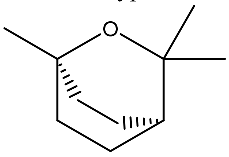
7 salvia officinalis					
59	Caffeic acid 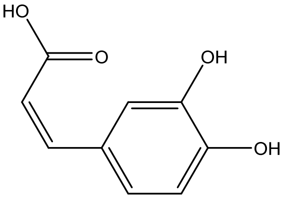	312	Beta-amirin 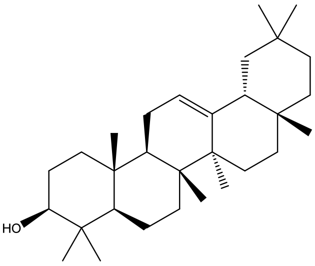	245	Quercetin 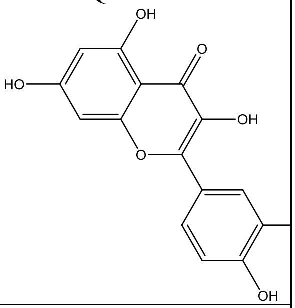
178	Luteolin 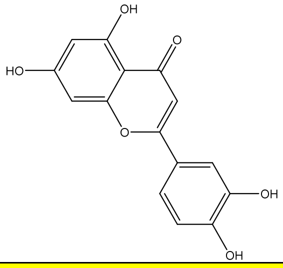	330	Beta-sitosterol 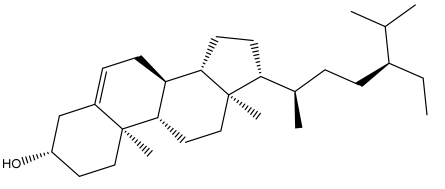		
8 marrubium Vulgare					

24	Acteoside 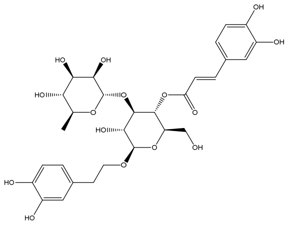	45	Ballotetroside 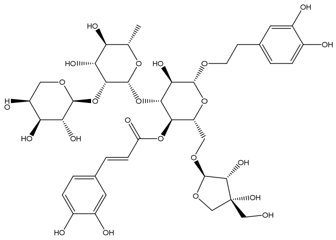	66	Carnosol 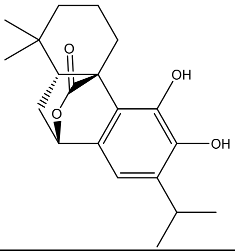
26	Aesculin 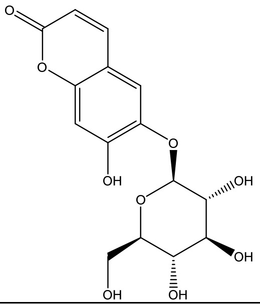	59	Caffeic acid 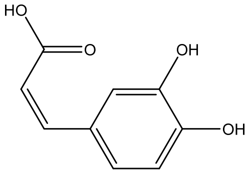	73	Chlorogenic acid 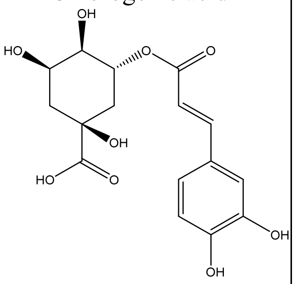
32	Alyssonoside 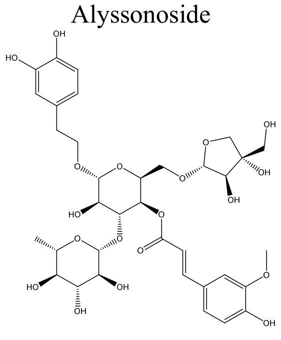	60	Caffeoylmalic acid 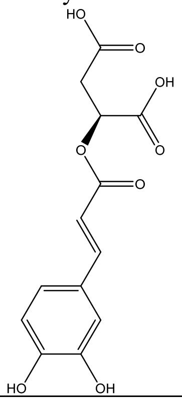	84	P-coumaric acid 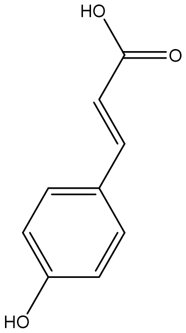
97	Deacetylvitexilactone 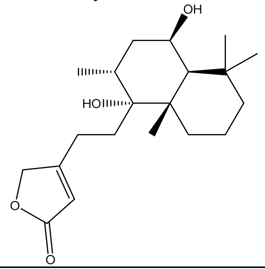	177	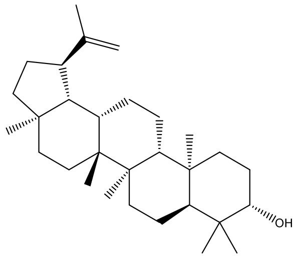 Lupeol	242	Protocatechuic acid 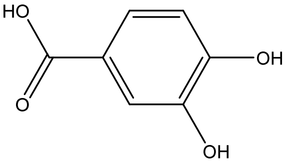
104	4-hydroxybenzoic acid 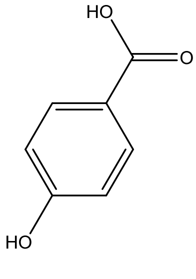	180	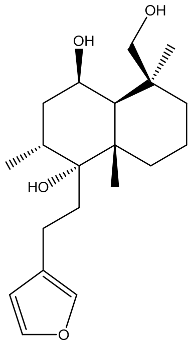 Marrubenol	253	Rosmarinic acid 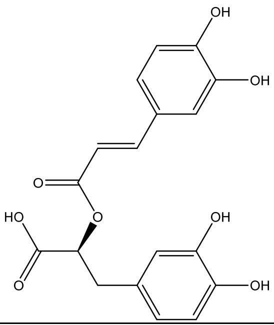

124	Ferulic acid 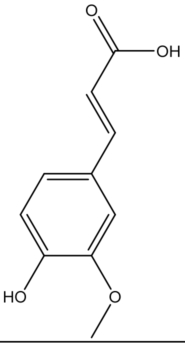	181	Marrubic acid 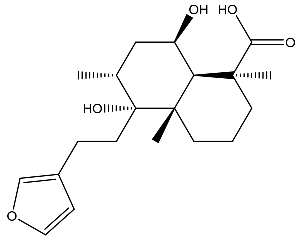	257	A sacranoside 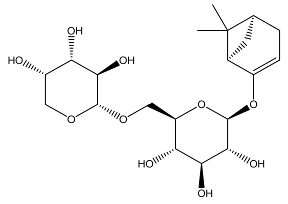
126	Forsythoside b 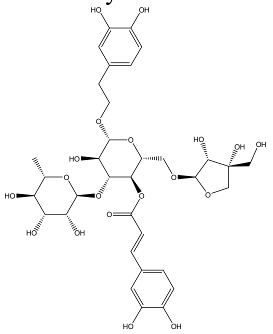	190	3-deoxo-15(s)-methoxyvelutine 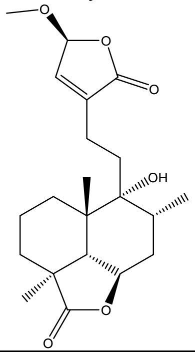	260	Samioside 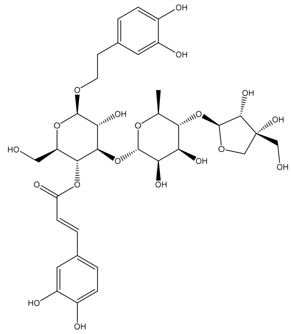
128	Gallic acid 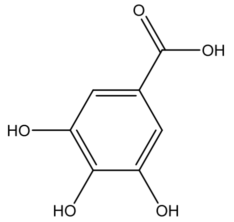	221	Oleanolic acid 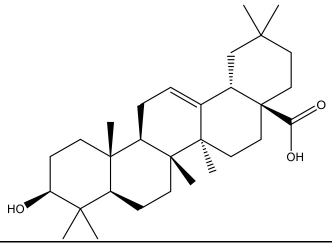	263	Sinapic acid 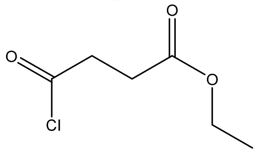
129	Gentisic acid 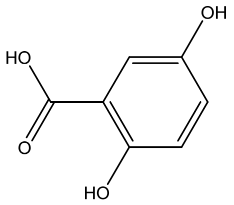	227	Peregrinin 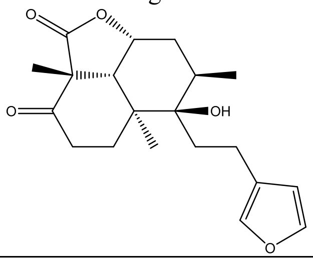	269	Syringic acid 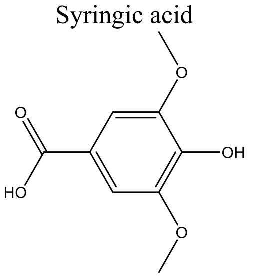

166	Leucosceptoside 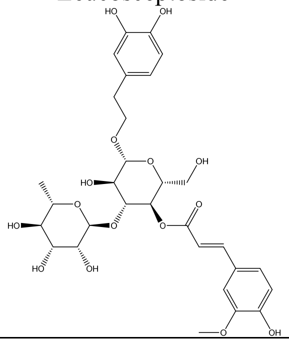	228	Peregrinol 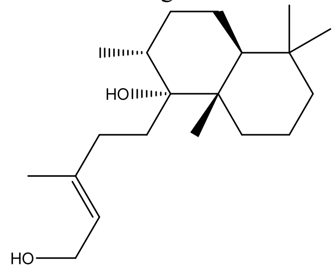	285	Umbelliferone 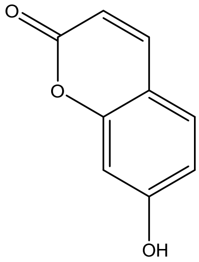
293	Vulgarin 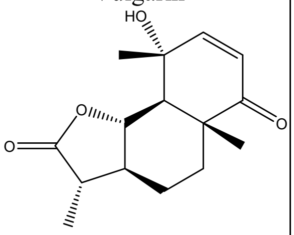	294	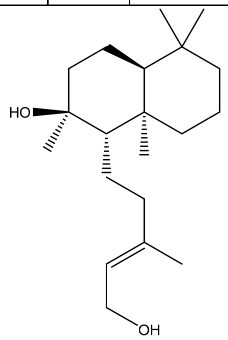 Vulgarol		
9 mentha spicata					
6	1-isobenzofuranone 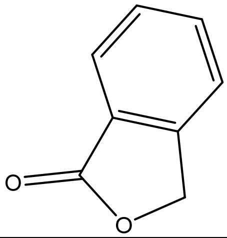	108	Eravacycline 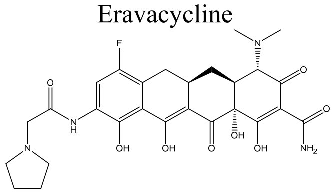	148	Hesperidin 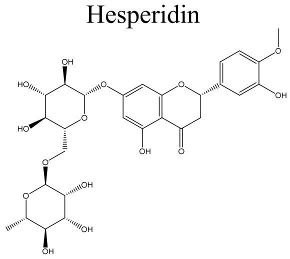
37	Apigenin-7-o-glucoside 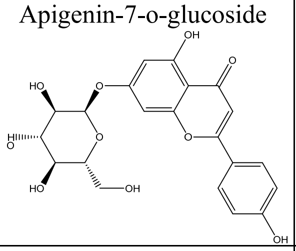	109	Eriodictyol-7-o-glucoside 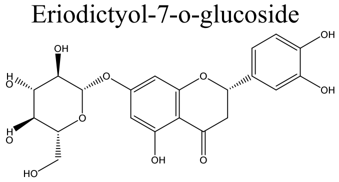	156	4-hydroxycoumarin 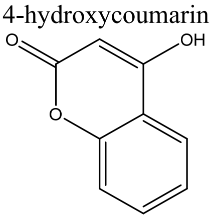
75	Chrysosplenetin 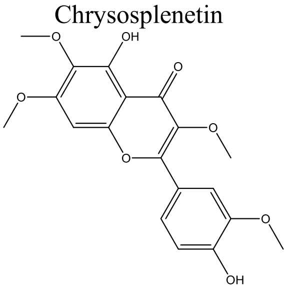	110	Erucic acid 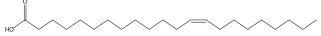	164	Kaempferol 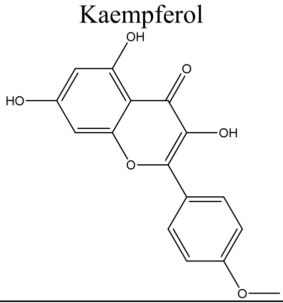

101	Decuroside iii<br>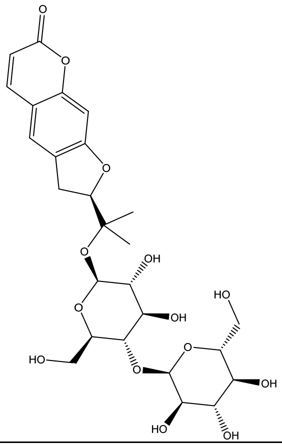	123	Ferreirin<br>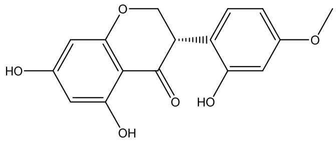	189	Meprednisone<br>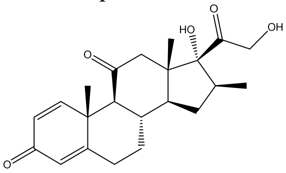
102	Demethylsulochrin<br>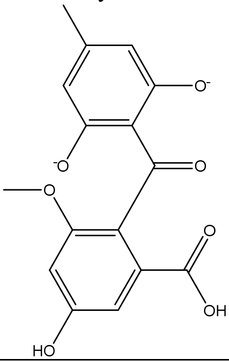	139	Ginkgolide<br>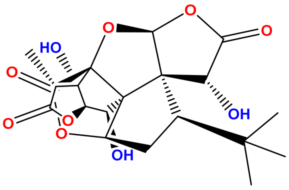	247	Retusin<br>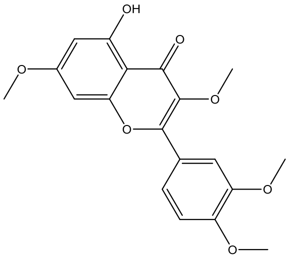
249	Rhamnocitrin<br>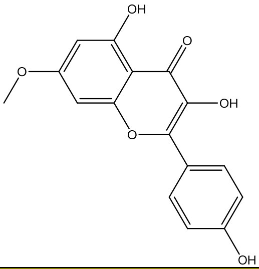	258	Safingol<br>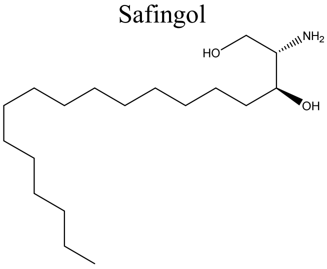	266	Stearamide<br>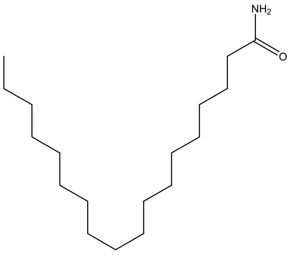
10 teucrium polium					
40	Aristolene<br>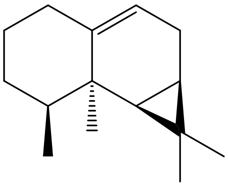	137	Germacrene-d-4-ol<br>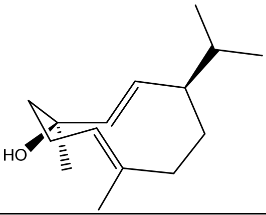	252	Rosifoliol<br>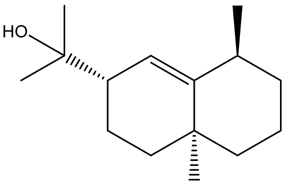

58	Cadinol 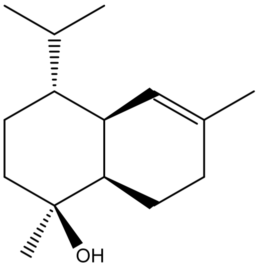	152	Himachalene 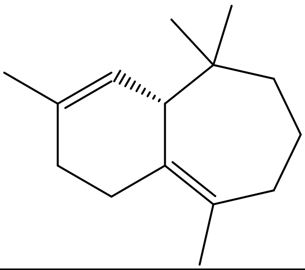	256	Sabinol 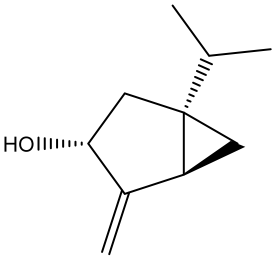
65	Carene 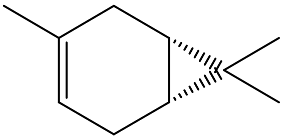	197	Alpha-muurolol 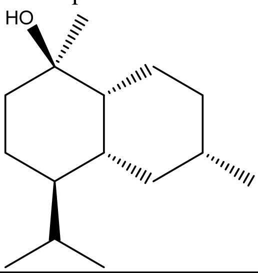	277	Thujene 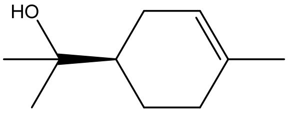
69	Caryophyllene 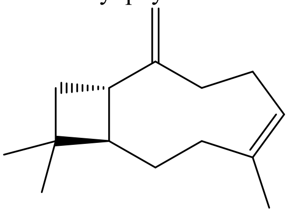	202	Myrtenol 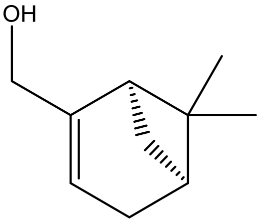	278	Thymol 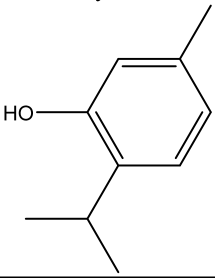
70	Caryophyllene oxide 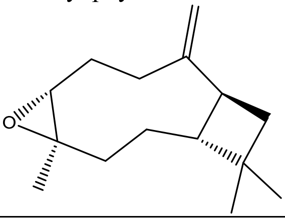	229	Phellandrene 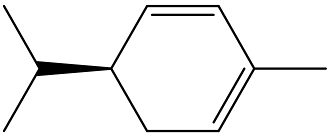	287	Valencene 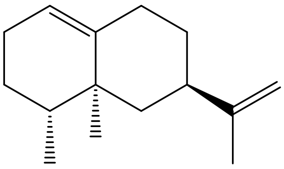
90	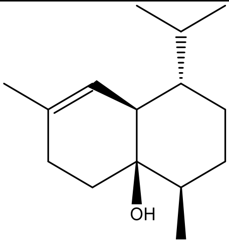 Cubenol				

<table>
<tr><td colspan="6">Rosaceae</td></tr>
<tr><td colspan="6">11 malus Domestica</td></tr>
<tr><td>73</td><td>Chlorogenic acid
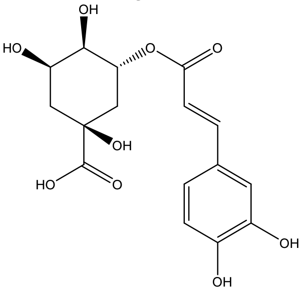</td><td>233</td><td>Phloretin
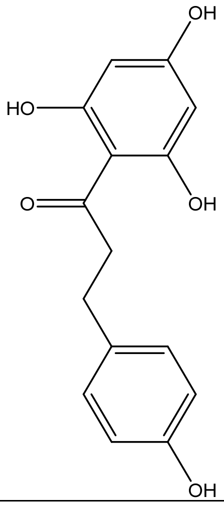</td><td>24
5</td><td>Quercetin
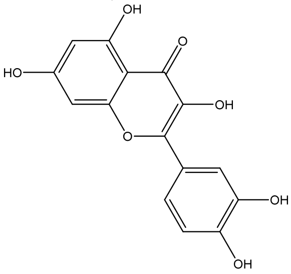</td></tr>
<tr><td>84</td><td>P-coumaric acid
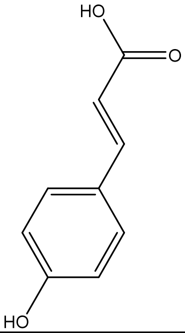</td><td>234</td><td>Phloridzin
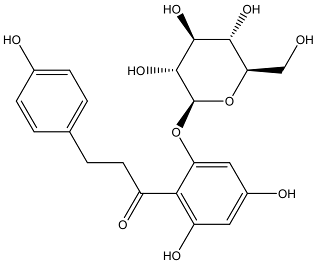</td><td>25
0</td><td>Quercetin-3-rhamnoside
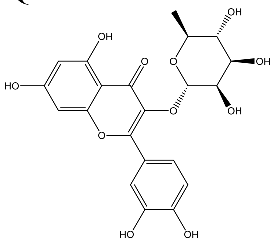</td></tr>
<tr><td>12
8</td><td>Gallic acid
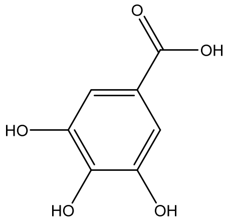</td><td>241</td><td colspan="3">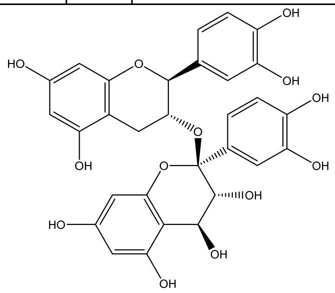
Procyanidin</td></tr>
<tr><td colspan="6">12 rosa Damascena</td></tr>
<tr><td>44</td><td>Astragalin
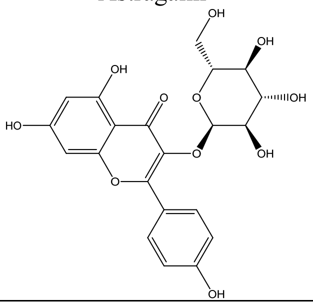</td><td>12
8</td><td>Gallic acid
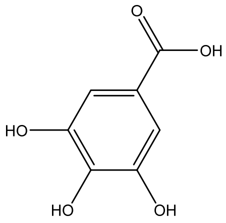</td><td>19
3</td><td>Methyl gallate
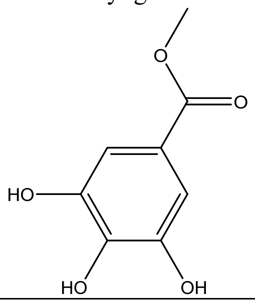</td></tr>
</table>

71	Catechin<br>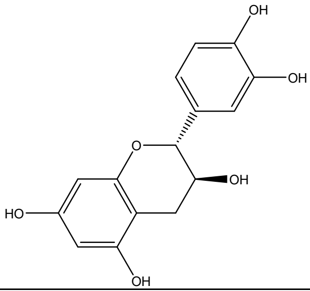	154	Phenethyl 3,4,5-trihydroxybenzoate<br>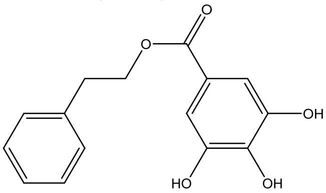	242	Protocatechuic acid<br>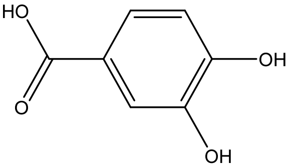
104	4-hydroxybenzoic acid<br>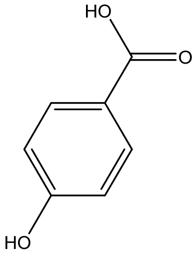	164	Kaempferol<br>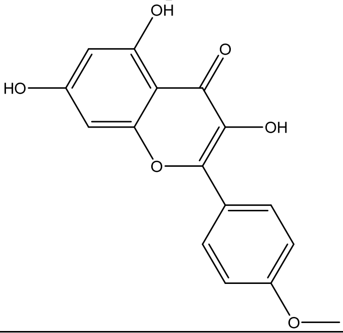	245	Quercetin<br>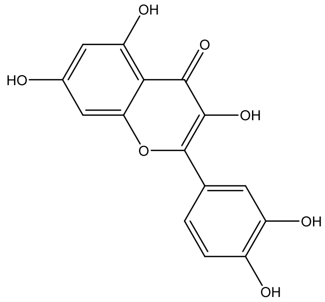

Zingiberaceae					
13 alpinia officinarum hance					
49	Bergamotol<br>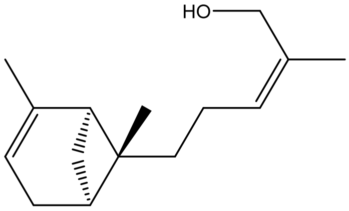	145	Alpha-gurjunene<br>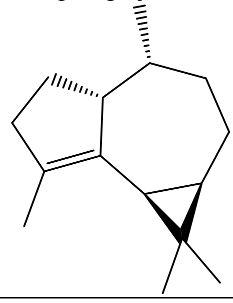	296	Alpha-bisabolol<br>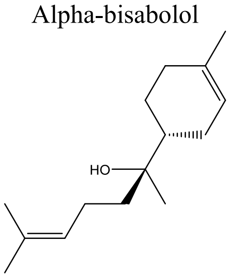
53	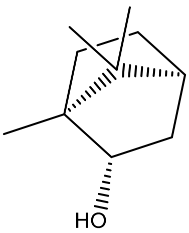<br>Borneol	157	Isobutyl benzoate<br>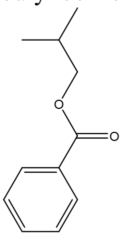	298	Alpha-calacorene<br>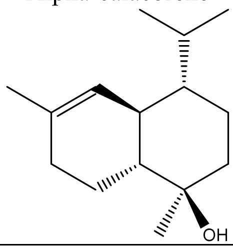
54	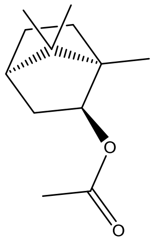<br>Bornyl acetate	159	Isocaryophillene<br>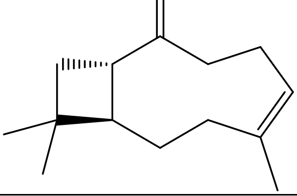	301	Alpha-farnesene<br>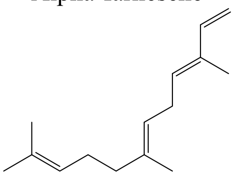

55	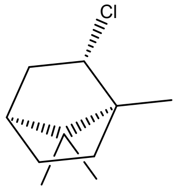 Bornyl chloride	196	Alpha-muurolene 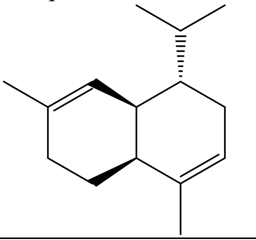	302	Alpha-guaiene 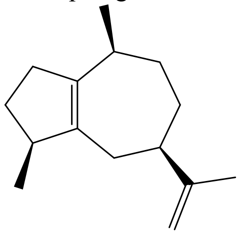
57	Butyric anhydride 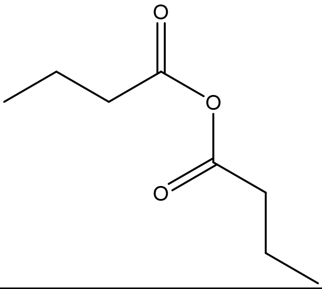	207	Nerolidol 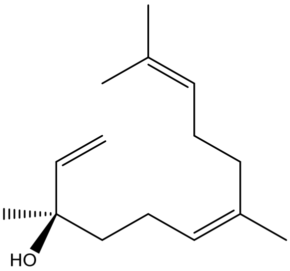	308	Alpha-selinene 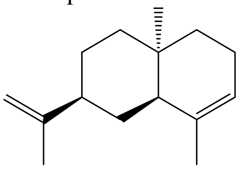
63	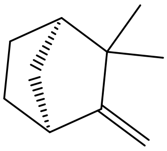 Camphene	230	Phenethyl isobutyrate 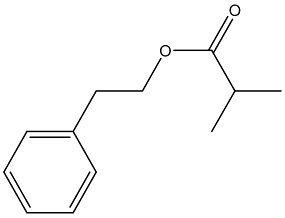	316	Beta-bisabolol 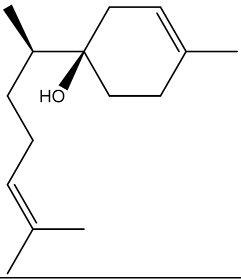
64	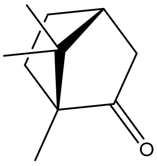 Camphor	231	Phenylethyl isovalerate 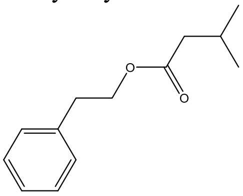	328	Beta-selinene 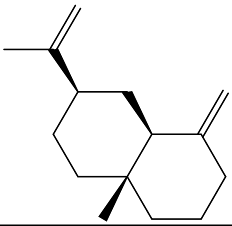
88	Alpha-selinene 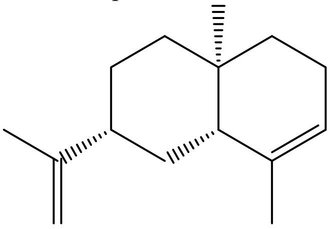	282	Beta-farnesene 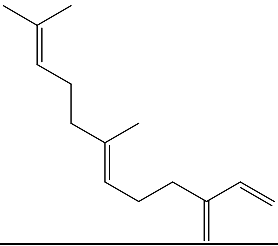	334	Γ-cadinene 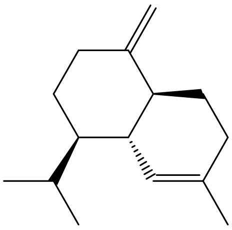
120	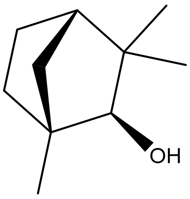 Fenchol	287	Valencene 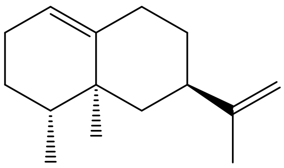	335	Γ-elemene 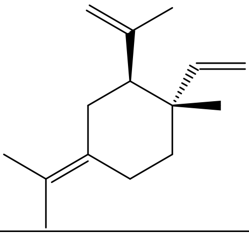

122	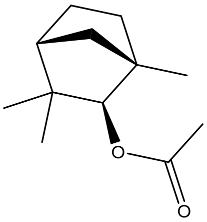 Fenchyl acetate	295	Alpha-bergamotene 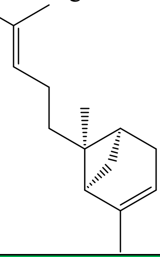	337	Δ-cadinene 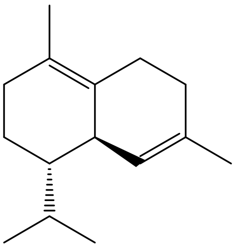
14 Curcuma longa					
14	2-methoxy-4-vinylphenol 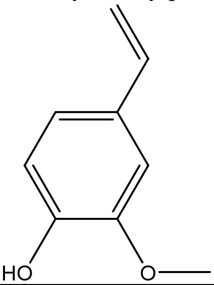	161	Isolongifolol 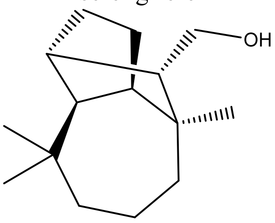	319	Beta-curcumene 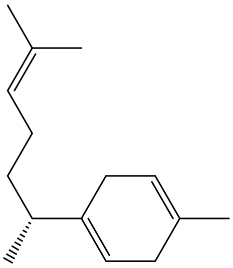
43	Ar-turmerone 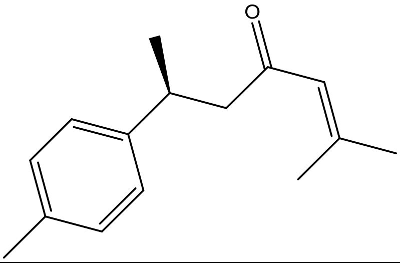	297	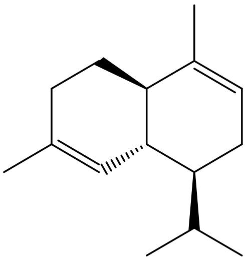 Alpha-cadinene		
127	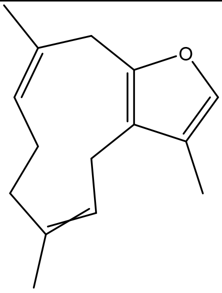 Furanodiene	299	Alpha-cedrene 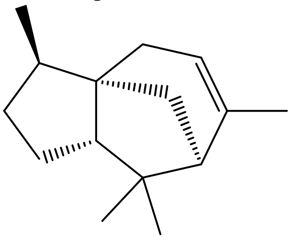	332	(+)-beta-turmerone 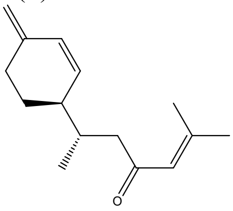
134	Geranyl-p-cymene 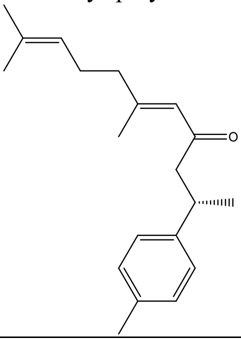	310	Alpha-turmerone 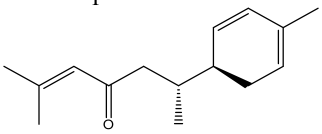	333	Beta-vatirenene 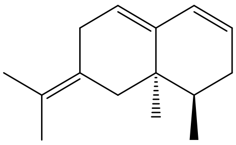

Apiaceae

15 foeniculum vulgare					
34	Anethole (trans cis) 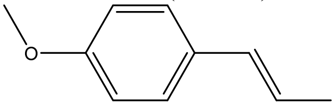	121	Fenchone 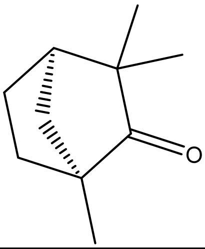	255	Sabinene 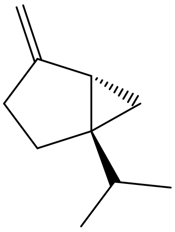
111	Estragole 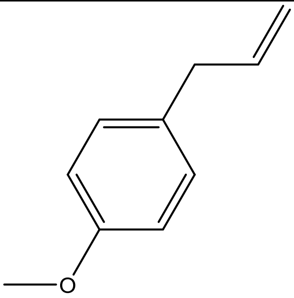	224	P-anisaldehyde 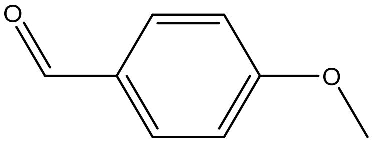		
16 Ammi visnaga					
8	1-tridecene 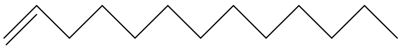	87	Croweacin 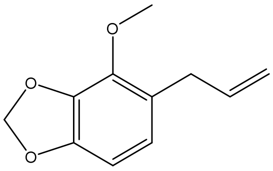	169	Limonene 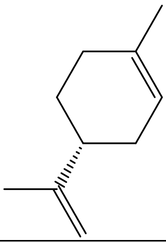
16	2-methyl propyl butanoate 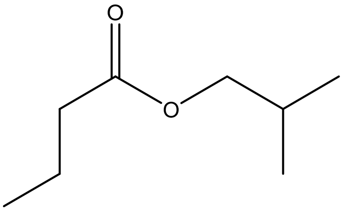	94	Cyclopentanol 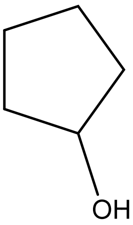	173	Linalyl butyrate 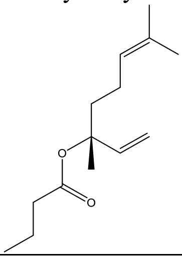
20	3,4,5-trimethoxybenzaldehyde 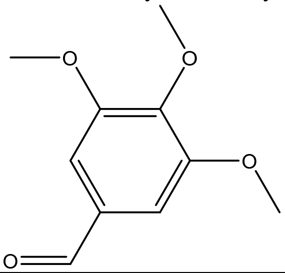	118	Eugenol 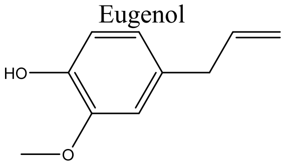	174	Linalyl valerate 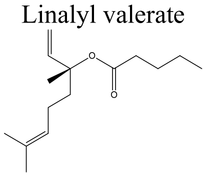

33	Amyl isobutyrate 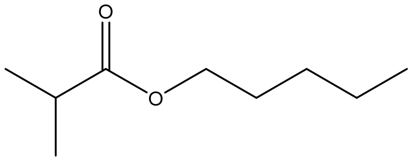	119	Farnesyl acetate 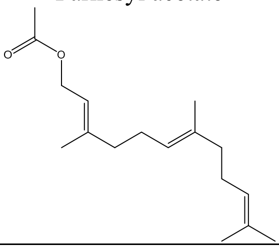	187	Menthol 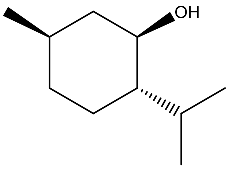
34	Anethole(trans cis) 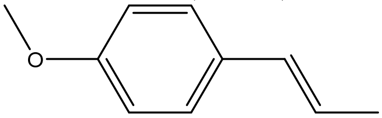	122	Fenchyl acetate 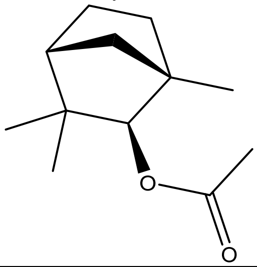	251	Rose oxide 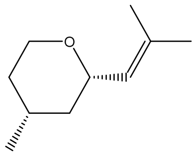
54	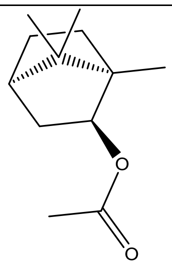 Bornyl acetate	132	Geranyl acetate 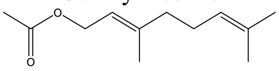	278	Thymol 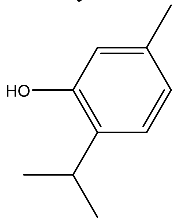
81	Citronellyl isobutyrate 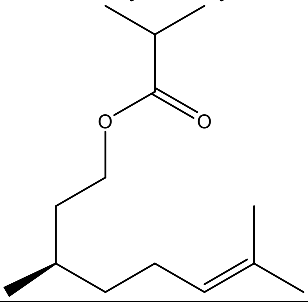	153	Humulene 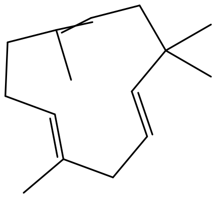	303	Alpha-isophorone 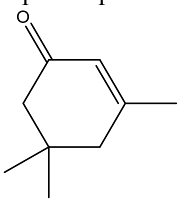
82	Citronellyl propionate 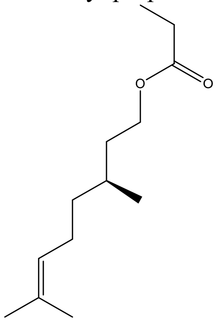	158	Isobutyl valerate 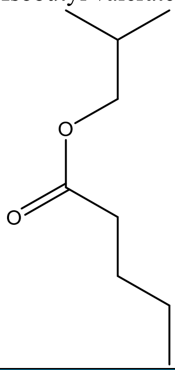	327	Beta-pinène 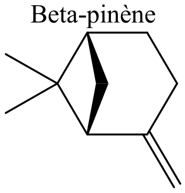
17 pimpinella anisum					

15	Pseudoisoeugenyl-2- methyl butyrate 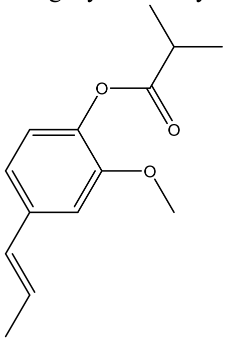	107	Epoxypseudoisoeugen yl-2-methylbutyrate 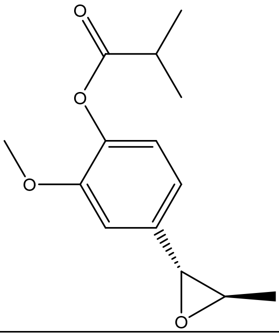	243	Pseudoisoeugenyl-2-methyl butyrate 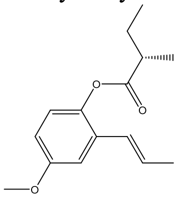
34	Anethole(trans cis) 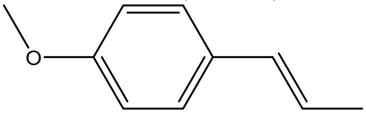	138	Geyrene 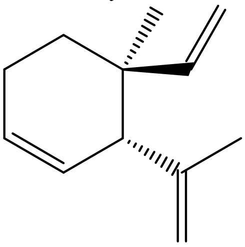	305	Alpha-phellandrene 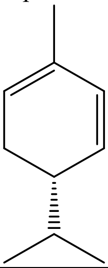
48	Epoxypseudoisoeugen yl-2-methylbutyrate 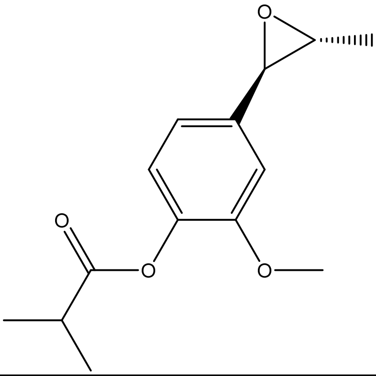	151	Hexatriacontane 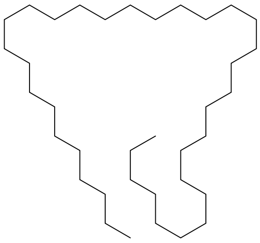	311	Alpha-zingiberene 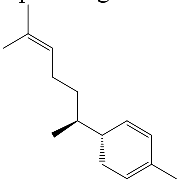
93	Cycloisolongifolene 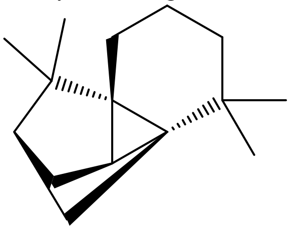	192	Methyl eugenol 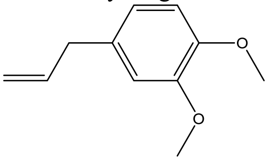	315	Beta-bisabolene 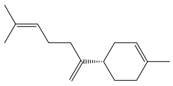

103	Docosane<br>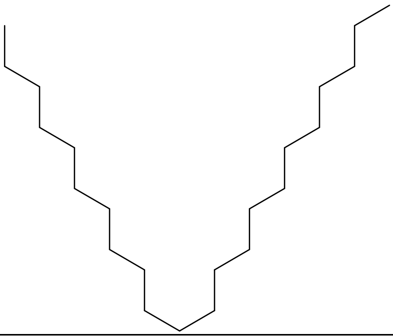	211	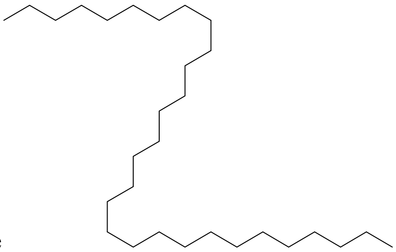<br>Nonacosane

Asteraceae					
18 artemisia herba alba					
12	2,5-bornanedione<br>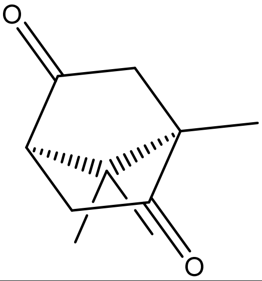	64	Camphor<br>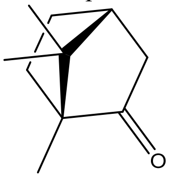	30<br>7	Alpha-santonin<br>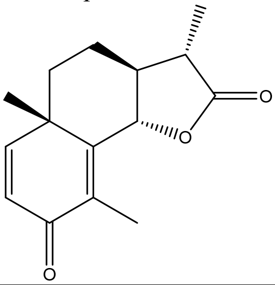
27	Alhanin<br>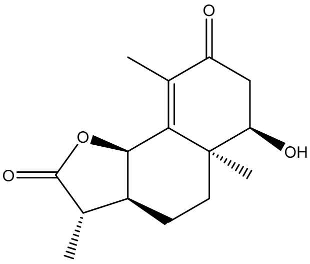	74	Chrysanthenone<br>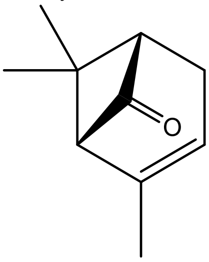	32<br>2	Germacrene b<br>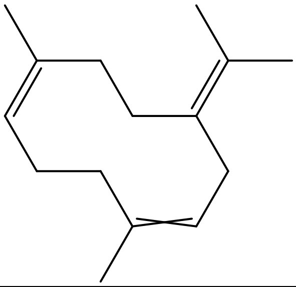
30	Alpha-thujone<br>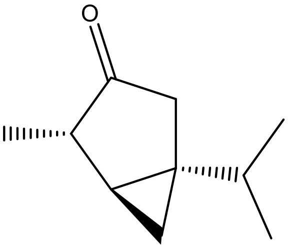	11<br>4	Eucalyptol<br>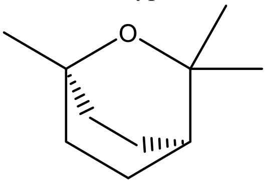	33<br>1	Beta-thujone<br>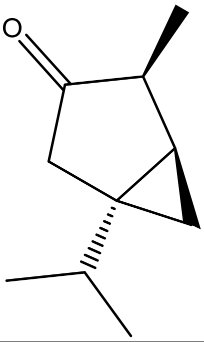
63	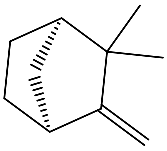<br>Camphene	27<br>3	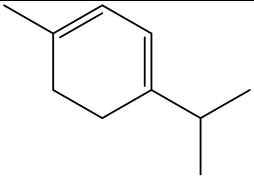<br>Alpha-terpinene		

19 Anacyclus pyrethrum					
72	8-cedren-13-ol acetate 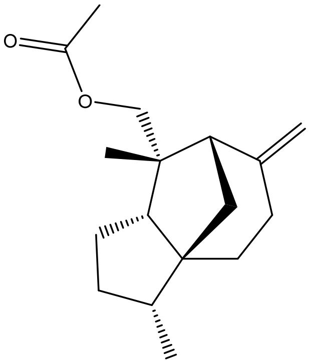	259	Salvial-4(14)-en-1-one 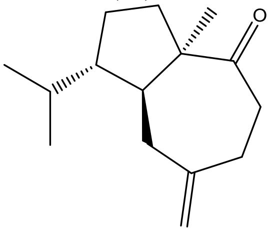	313	Beta-atlantol 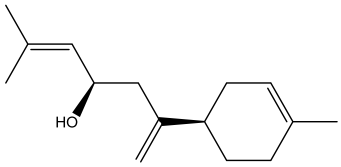
115	[alpha]-eudesmol acetate 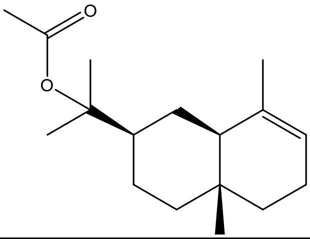	281	Trans-isolongifolanone 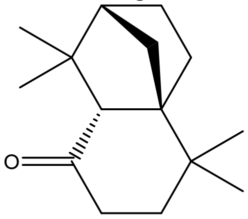	314	Beta-biotol 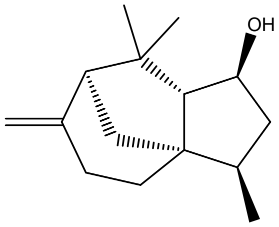
196	Alpha-muurolene 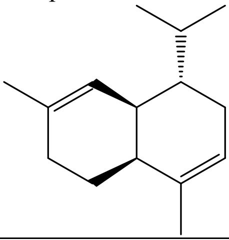	290	Vetivenic acid 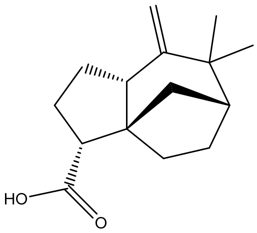	317	Beta-copaen-4- alpha-ol 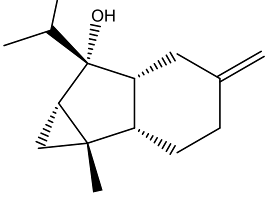
217	Occidentalol 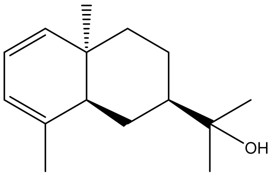	304	Alpha-neocallitropsene 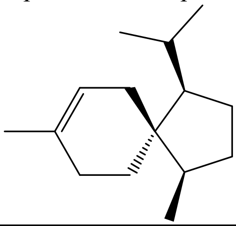	325	Beta-ionone 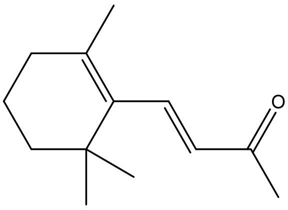
20 matricaria camomilla					
36	Apigenin 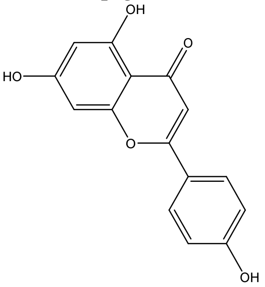	73	Chlorogenic acid 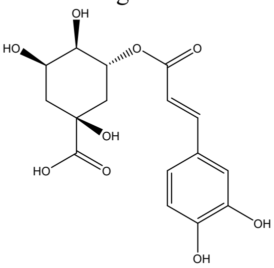	245	Quercetin 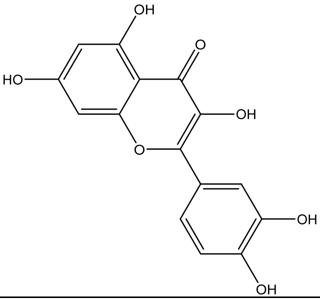

37	Apigenin-7-o-glucoside<br>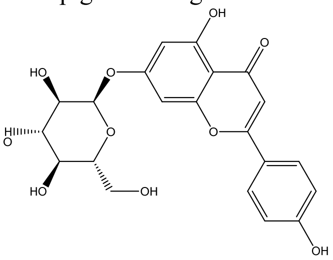	12<br>4	Ferulic acid<br>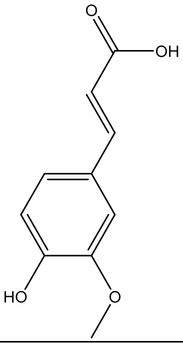	27<br>9	Trans-β-ionone<br>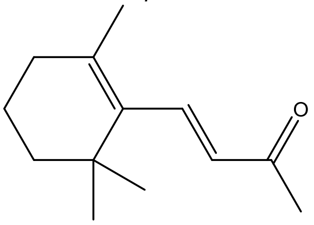
59	Caffeic acid<br>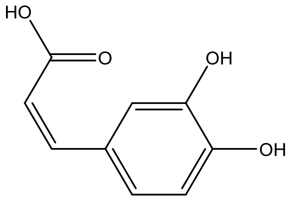	17<br>8	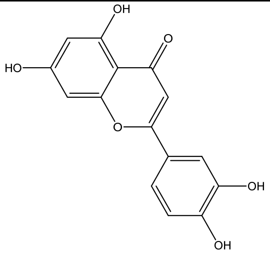<br>Luteolin		

Lauraceae					
21 Cinnamomum burmanni					
85	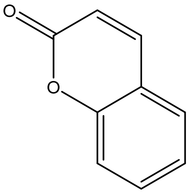<br>Coumarin	26<br>9	Syringic acid<br>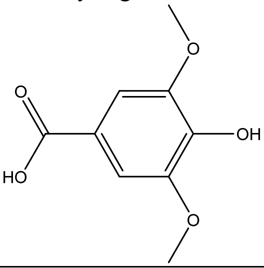	16<br>4	Kaempferol<br>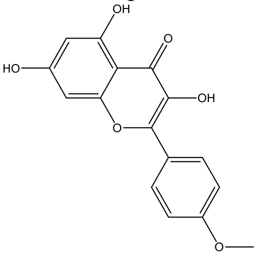
10<br>4	4-hydroxybenzoic acid<br>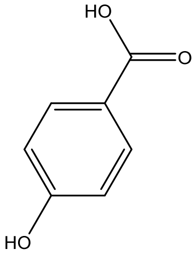	28<br>8	Vanillic acid<br>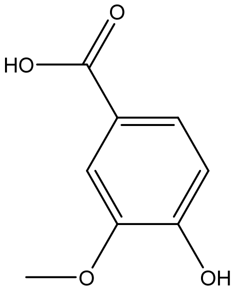	19<br>4	Methyl vanillate<br>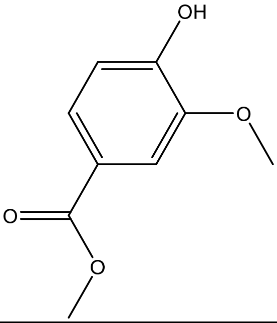
12<br>5	Ficaprenol-10<br>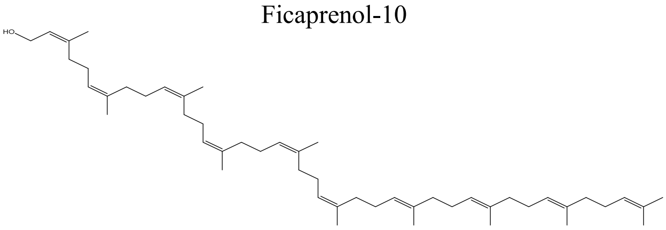			26<br>5	Squalene<br>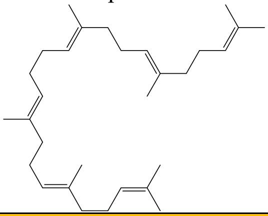
22 Laurus Nobilis					

51	Bicyclogermacrene 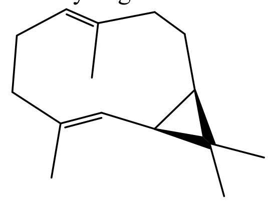	208	N-heptacosane 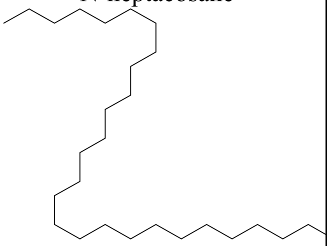	216	N-tricosane 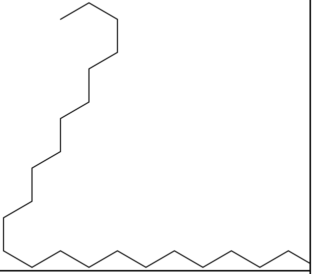
54	Bornyl acetate 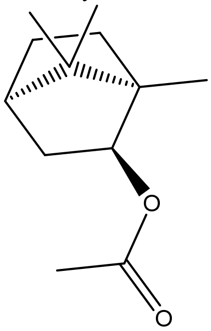	209	N-hexacosane 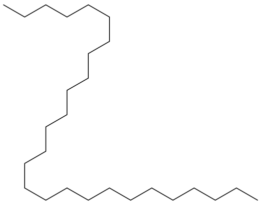	235	Phytol 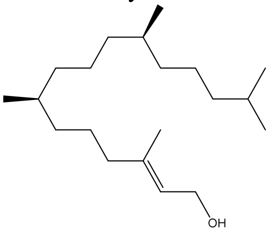
63	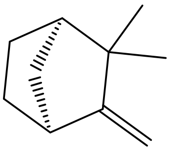 Camphene	210	N-octacosane 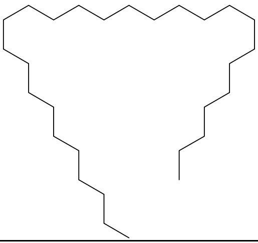	265	Squalene 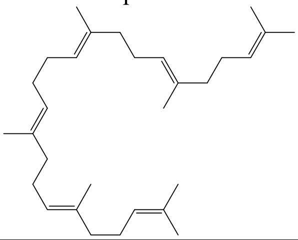
71	Catechin 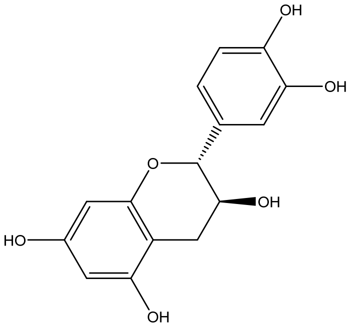	214	N-pentacosane 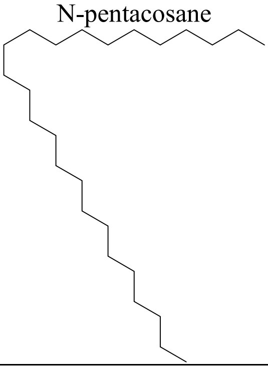	321	Beta-elemene 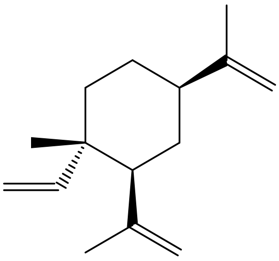
204	N-docosane 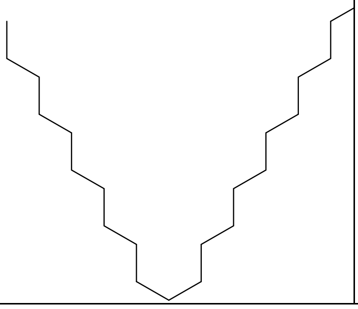	215	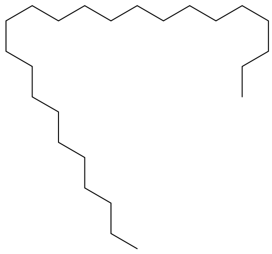 N-tetracosane		

Fabaceae					
23 cassia absus					
29	Alpha-linolenic acid 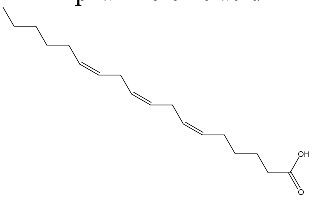	168	Lignoceric acid 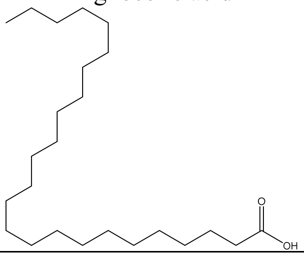	222	Oleic acid 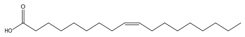
39	Arachidic acid 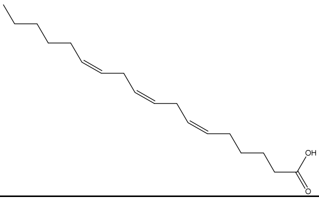	179	Margaric acid 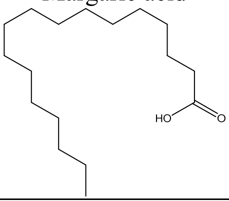	223	Palmitic acid 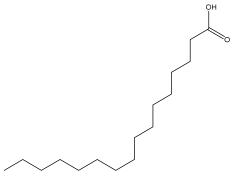
46	Behenic acid 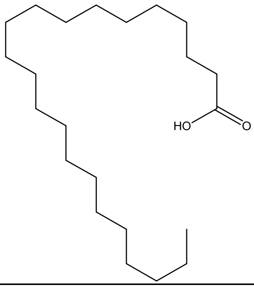	201	Myristic acid 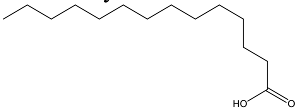	267	Stearic acid 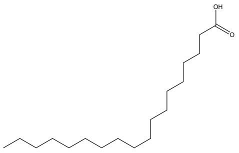
105	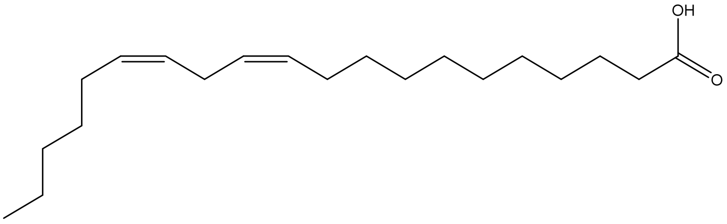 Eicosadienoic acid				
24 acacia raddiana					
1	(e)-2-heptenal 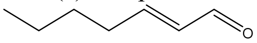	79	Citronellol 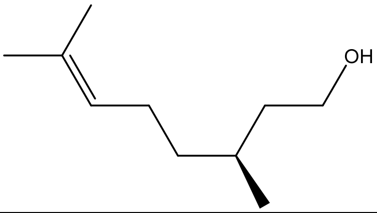	212	Nonanal 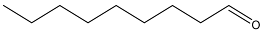

2	(e)-2-octenal 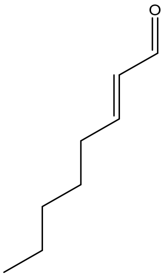	80	Citronellyl acetate 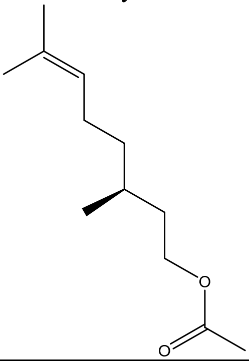	21<br>3	Nonanoic acid 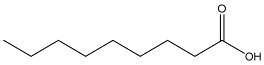
3	(e)-cinnamaldehyde 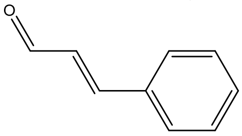	98	Decanal 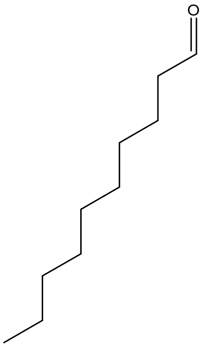	23<br>7	Piperitone 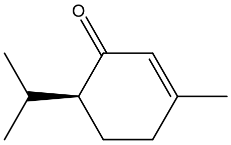
10	2,4-nonadienal 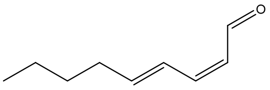	99	Decanoic acid 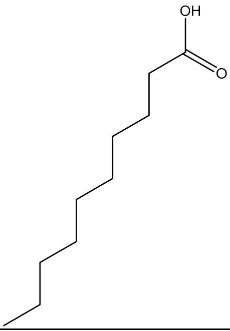	23<br>8	P-menth-9-en-1-ol 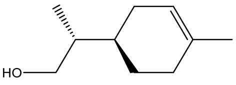
17	2-nonanone 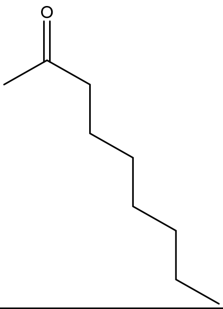	11<br>2	Ethyl 2, 4-decadienoate 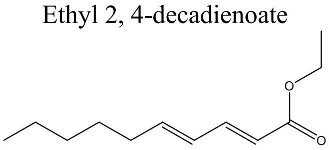	28<br>0	Trans-carveol 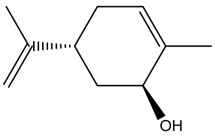

18	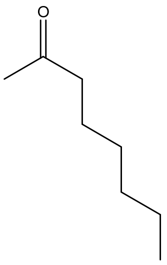 2-octanone	113	Ethyl decanoate 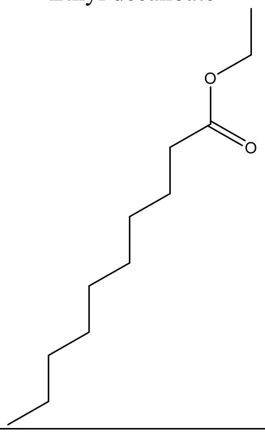	283	Tridecane
19	2-phenyl ethyl benzoate 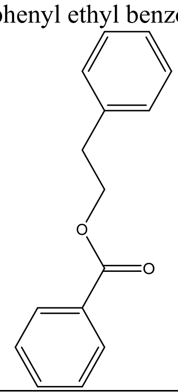	130	Geraniol 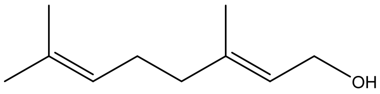	284	Tridecanol 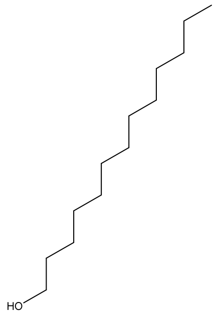
23	Acetophenone 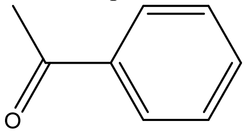	133	Geranyl acetone 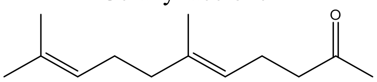	320	Beta-cyclocitral 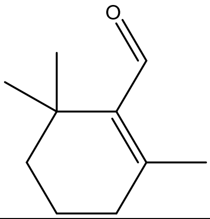
47	Benzyl benzoate 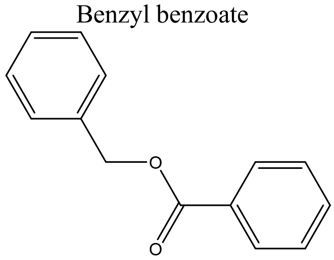	147	Heptanal 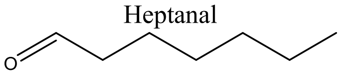	324	Beta-guaiene 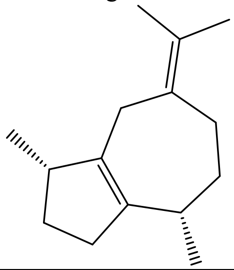

56	Bulnesol 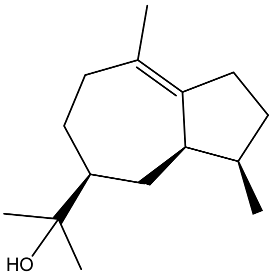	149	Hexadecane 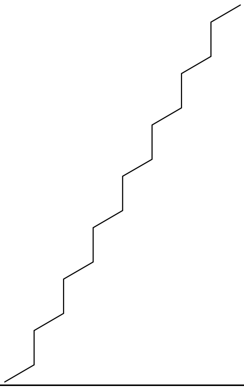	325	Beta-ionone 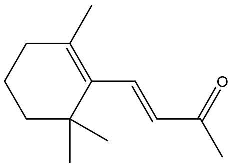
61	Calamenene 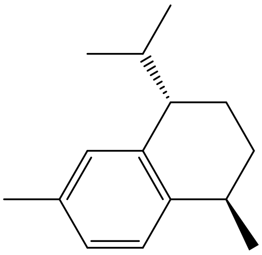	160	Isoelemicin 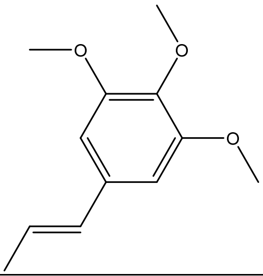	334	Γ-cadinene 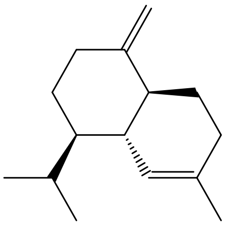
68	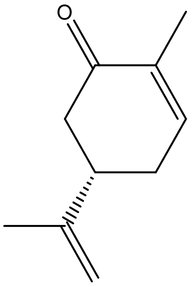 Carvone	191	Methyl cinnamate 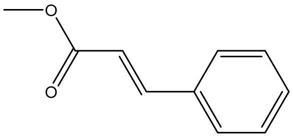	335	Γ-elemene 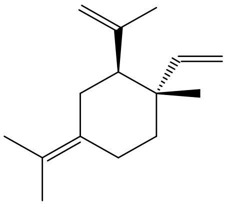
71	Catechin 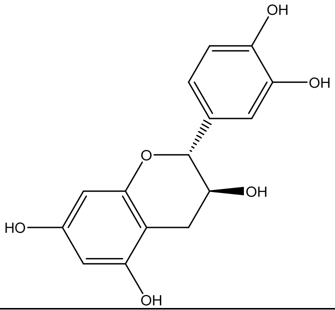	192	Methyl eugenol 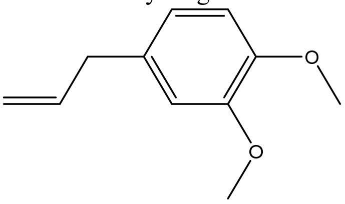	338	Δ-elemene 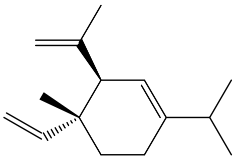
76	Cinnamic acid 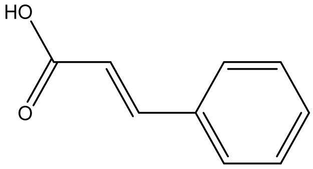	207	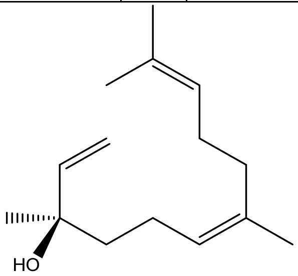 Nerolidol		
25 glycyrrhiza glabra					

4	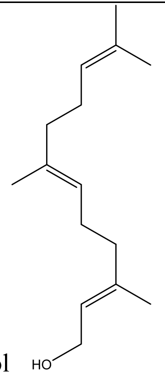 (e,e)-farnesol	14 3	Glycyrrhetic acid 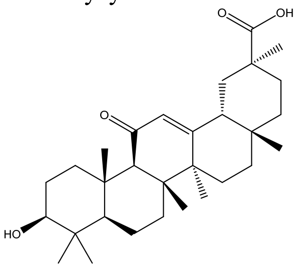	17 6	Liquiritin 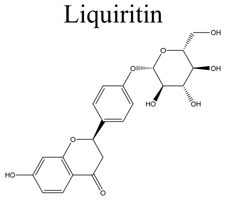
38	Apioside 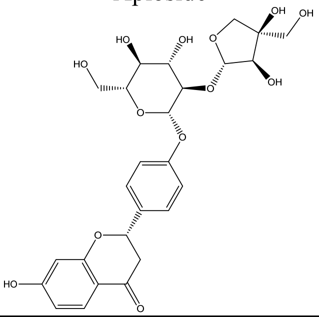	14 4	Glycyrrhizin 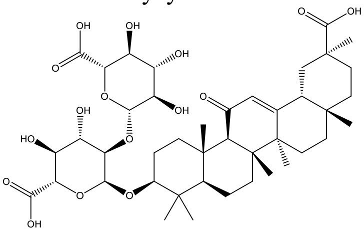	32 3	Beta-glycyrrhetinic acid 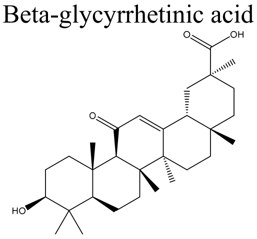
10 0	Decanol 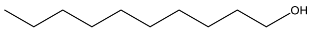	16 7	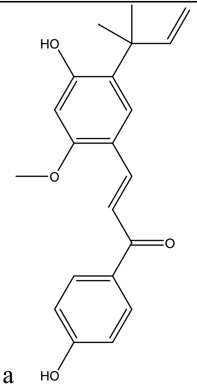 Licochalcone a	17 6	Liquiritin 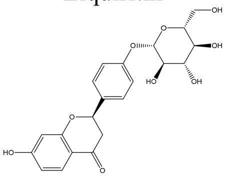
14 0	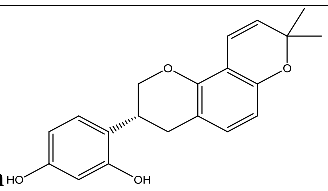 Glabridin				

Myrtaceae					
26 syzygium aromaticum					
62	Campesterol 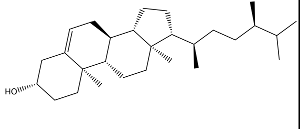	86	Crategolic acid 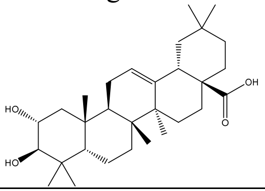	11 7	Eugenin 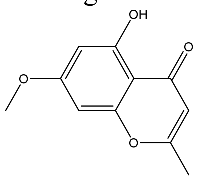

118	Eugenol 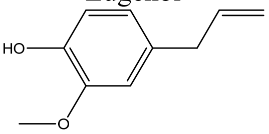	128	Gallic acid 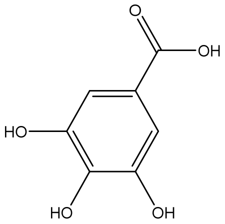	164	Kaempferol 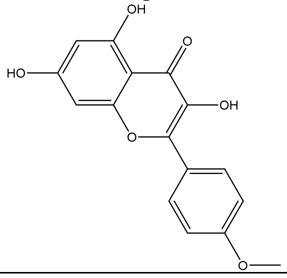
200	Myricetin 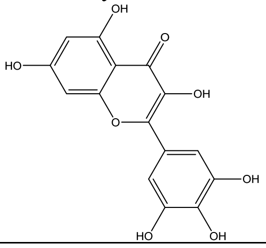	268	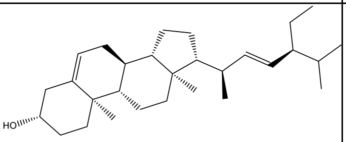 Stigmasterol	221	Oleanolic acid 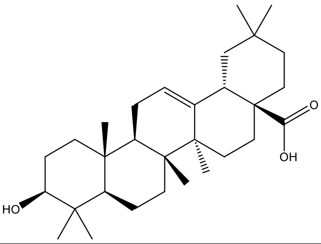
289	Vanillin 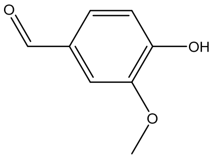	50	bicornin 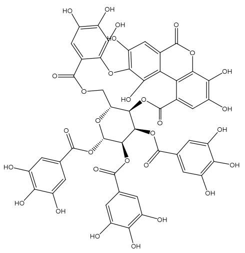	52	Biflorin 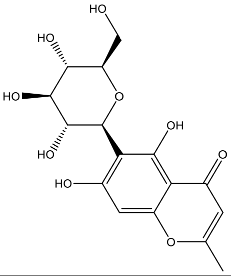
248	Rhamnetin 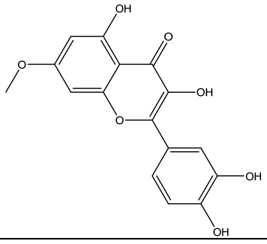				
27 Myrtus communis					
70	Caryophyllene, oxide 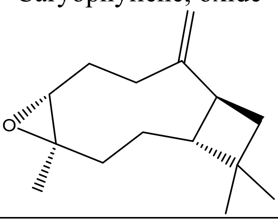	130	geraniol 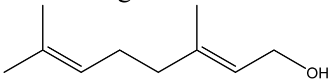	132	Geranyl acetate 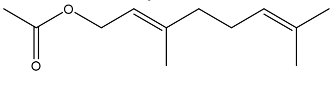
170	Linalool 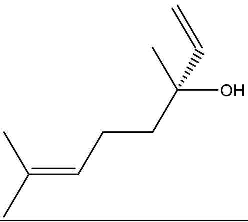	192	methyl eugenol 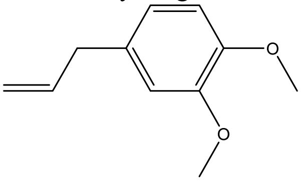	199	myrcene 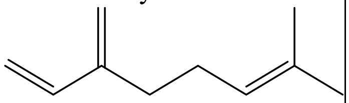

203	myrtenyl acetate 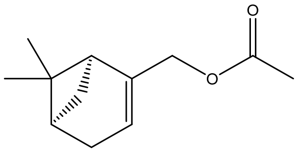	274	Terpineol 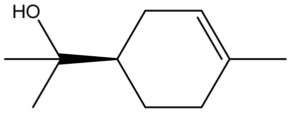	306	alpha-pinene 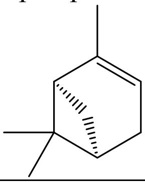
28 eucalyptus globulus					
63	Camphene 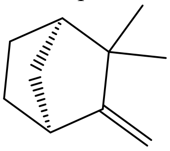	68	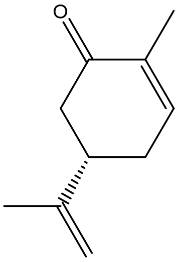 Carvone	77	Citral 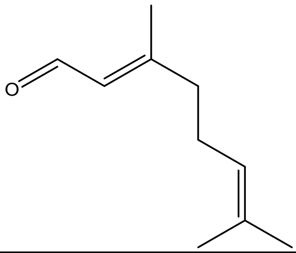
78	Citronellal 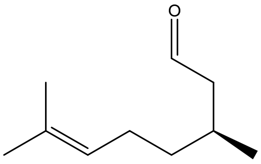	132	Geranyl acetate 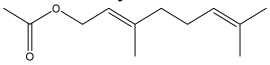	169	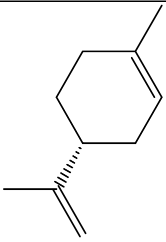 Limonene
170	Linalool 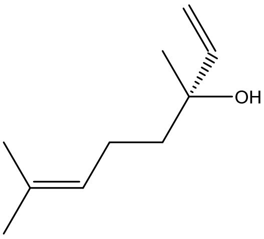	270	Tereticornate 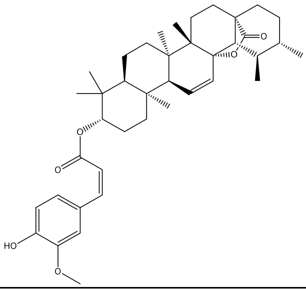		

Table S2: The 2D structure of the selected compounds after molecular docking simulation.

**Salvia officinalis**		
Luteolin L22 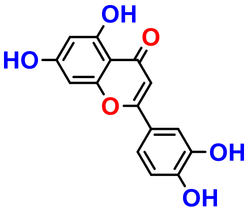	Quercetin L36 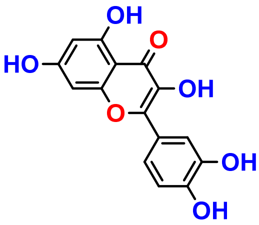	
**Mentha spicata**		

<table>
<tr><td>L5 Apigenin-7-o-glucoside
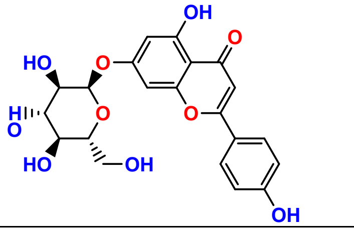</td><td>L3 Eriodictyol-7-o-glucoside
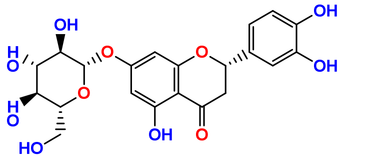</td><td>L35 Rhamnocitrin
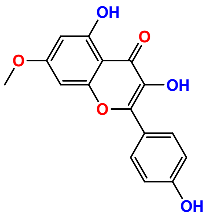</td></tr>
<tr><td>L2 Eravacycline
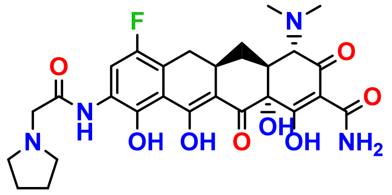</td><td>L9 Decuroside iii
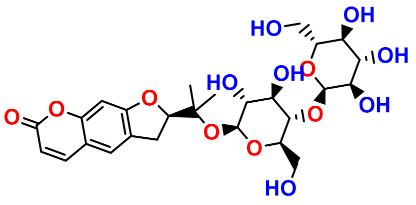</td><td>L18 Ginkgolide
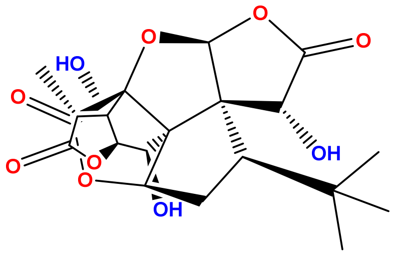</td></tr>
<tr><td>L11 Hesperidin
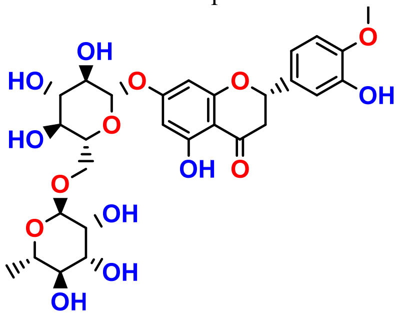</td><td>L40 Ferreirin
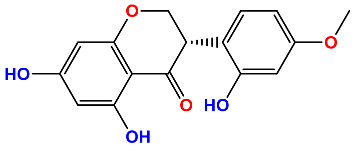</td><td>L10 Retusin
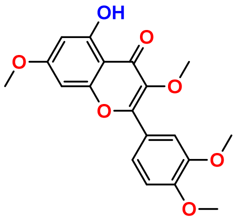</td></tr>
<tr><td>L32 Kaempferol
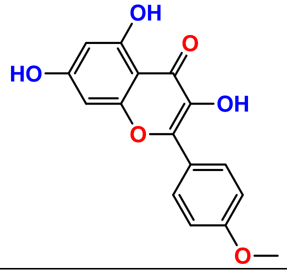</td><td>L20 Meprednisone
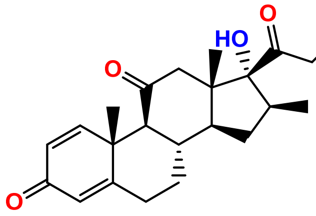</td><td></td></tr>
<tr><td colspan="3">Rosaceae malus Domestica</td></tr>
<tr><td>L12 Quercetin-3-rhamnoside
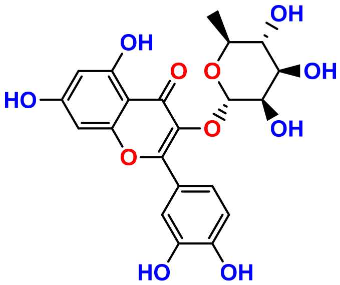</td><td>L38 Phloridzin
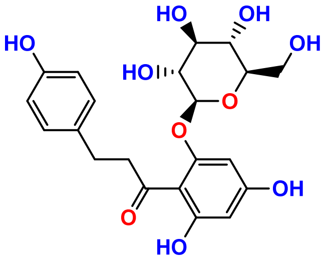</td><td></td></tr>
</table>

<table>
<tr><th colspan="3">Rosaceae rosa Damascena</th></tr>
<tr><td>L29 Astragalin
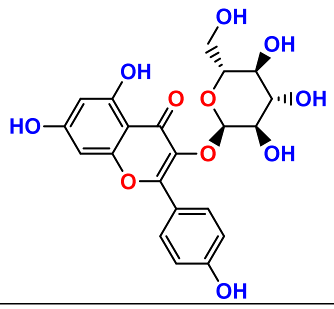</td><td></td><td></td></tr>
<tr><th colspan="3">Asteraceae artemisia herba alba</th></tr>
<tr><td>L28 Alhanin
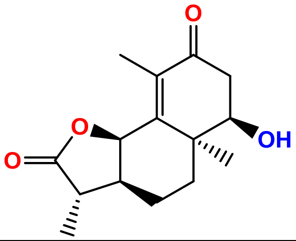</td><td>L33 Alpha-santonin
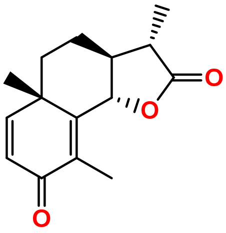</td><td></td></tr>
<tr><th colspan="3">Asteraceae matricaria camomilla</th></tr>
<tr><td>L26 Apigenin
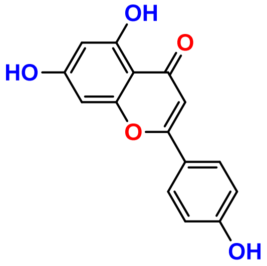</td><td></td><td></td></tr>
<tr><th colspan="3">Acacia raddiana</th></tr>
<tr><td>L39 Beta-guaiene
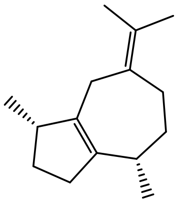</td><td></td><td></td></tr>
<tr><th colspan="3">Glycyrrhiza glabra</th></tr>
</table>

<table>
<tr><td>L1 Beta-glycyrrhetinic acid
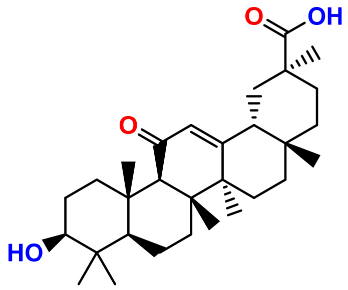</td><td>L24 Licochalcone a
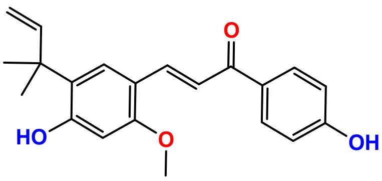</td><td>L13 Liquiritin
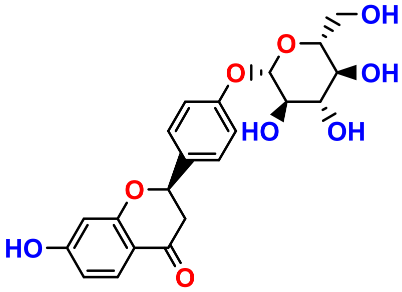</td></tr>
<tr><td>L6 Glabridin
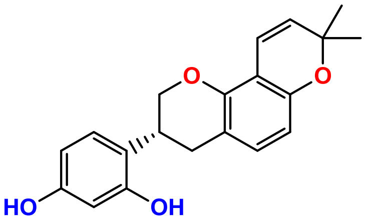</td><td></td><td></td></tr>
<tr><td colspan="3">Myrtaceae syzygium aromaticum</td></tr>
<tr><td>L17 Campesterol
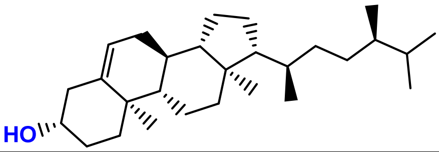</td><td></td><td></td></tr>
<tr><td>L27 Rhamnetin
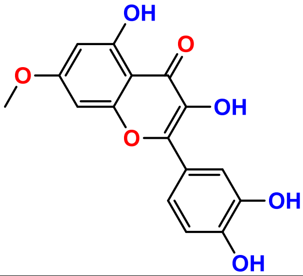</td><td>L7 Stigmasterol
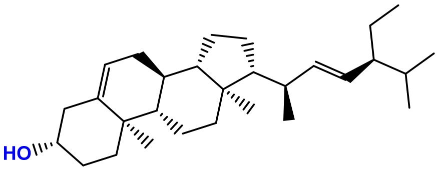</td><td></td></tr>
<tr><td colspan="3">Marrubium Vulgare</td></tr>
</table>

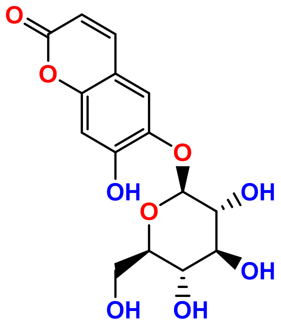 L23 Aesculin	L8 Alyssonoside 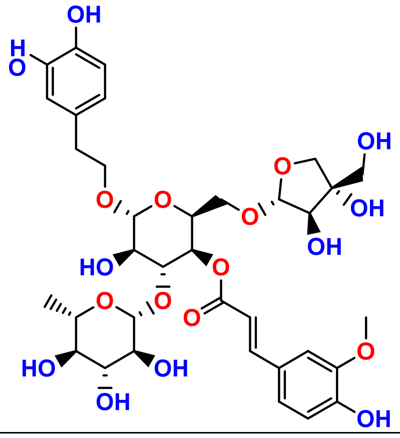	L21 carnosol 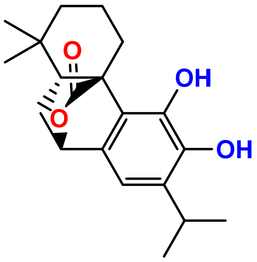
L31 Marrubenol 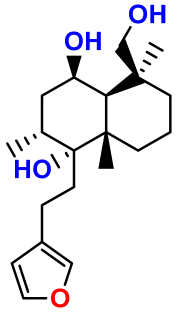	L34 Rosmarinic acid 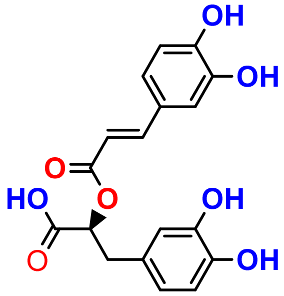	L25 Peregrinin 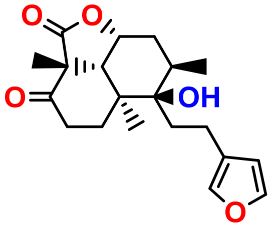
L4 Forsythoside b 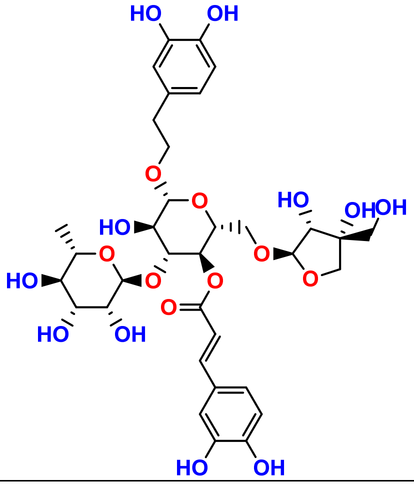	L19 3-deoxo-15(s)-methoxyvelutine 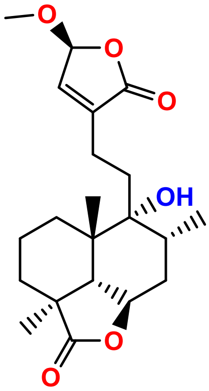	

L14 Leucosceptoside 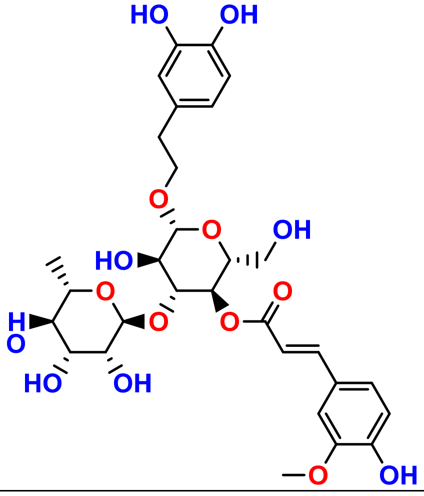	L37 Vulgarin 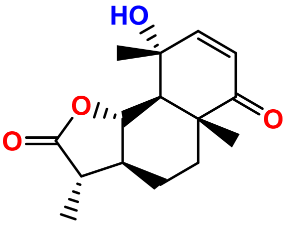	L15 Deacetylvitexilactone 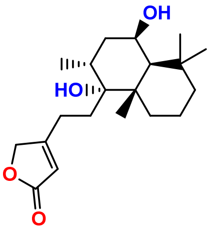
L30 Marrubic acid 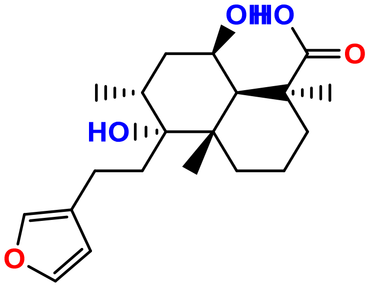	L16 Samioside 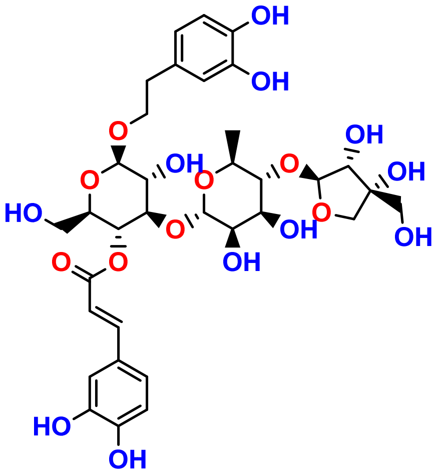	
